# Beyond conservative management: Surgical excision for symptomatic Sinding-Larsen–Johansson syndrome: A case report and literature review

**DOI:** 10.1097/MD.0000000000046792

**Published:** 2025-12-26

**Authors:** Hamzah A. Alhamzah, Demah M. Benfaris, Zyad A. Aldosari, Abdulnasser A. Alwabel, Mohannad W. Awwad

**Affiliations:** a Department of Orthopedic Surgery, College of Medicine, King Saud University, Riyadh, Saudi Arabia; b College of Medicine, King Saud University, Riyadh, Saudi Arabia.

**Keywords:** anterior knee pain, bone wax, osteochondrosis, patella, Sinding-Larsen–Johansson syndrome

## Abstract

**Rationale:**

Sinding–Larsen–Johansson syndrome (SLJS) is an osteochondrosis of the inferior patellar pole that typically affects adolescents and usually resolves with conservative treatment. Persistence into adulthood is rare, particularly when associated with symptomatic ossicle formation, and the optimal management of such cases remains uncertain.

**Patient concerns:**

A 21-year-old male recreational basketball player presented with chronic anterior knee pain persisting for 6 years, aggravated by kneeling and jumping, despite prior extensive conservative management during adolescence.

**Diagnoses:**

Clinical examination revealed a firm, mobile swelling distal to the patella, with preserved motion and knee stability. Serial imaging demonstrated progression to a discrete ossicle at the inferior patellar pole, consistent with stage IV-B SLJS.

**Interventions:**

Surgical excision of the symptomatic ossicle was performed through a midline approach with minimal patellar tendon disruption. Bone wax was applied to the exposed cancellous surface of the patella.

**Outcomes:**

Postoperative recovery was uneventful. The patient achieved early mobilization, regained full function by 1 month, and experienced complete resolution of symptoms. Histopathology confirmed benign mature bone.

**Lessons:**

This case demonstrates that adult persistence of SLJS with ossicle formation, though exceptionally rare, may require surgical excision when conservative therapy fails. Careful resection with preservation of the extensor mechanism can provide reliable pain relief and restoration of full function.

## 1. Introduction

Sinding-Larsen–Johansson syndrome (SLJS) is a traction related osteochondrosis that affects the inferior pole of the patella,^[1]^ 1st described in 1921 by Sinding-Larsen and Johansson as pain localized to the distal patella with radiographic evidence of pole fragmentation.^[2]^ The condition is thought to stem from repetitive traction forces at the origin of the patellar tendon, which attaches to the still-cartilaginous inferior pole in adolescents. Increased quadriceps tension and repetitive loading, particularly in active youth engaged in jumping sports, can lead to swelling of the cartilage, calcification, tendon thickening, and even partial fragmentation of the patella. In some cases, SLJS may also be associated with infrapatellar bursitis.^[3]^ Medlar and Lyne later proposed that the pathophysiology involves traction tendinitis with de novo calcification at the proximal insertion of the patellar tendon, often with partial avulsion.^[4]^ SLJS most commonly presents in adolescent males aged 10 to 14 and accounts for approximately 2% to 5% of cases of anterior knee pain in this group.^[5]^ However, its occurrence in adults is extremely rare and poorly documented in the literature, especially when calcific changes persist and become symptomatic beyond skeletal maturity.

The available literature on SLJS is predominantly limited to case reports, with few detailed or standardized treatment protocols. Nevertheless, conservative management typically involving activity restriction or modification, immobilization or bracing, nonsteroidal anti-inflammatory drugs, physical therapy including muscle stretching and strengthening, and gradual return to sport has been the cornerstone of treatment. Recovery times vary widely, ranging from a few weeks to several months, reflecting differences in clinical presentation, adherence, and possibly diagnosis.^[6]^ In this report, we present a rare case of SLJS in a 21-year-old recreational basketball player and culinary student. We describe the surgical management approach, including excision of the calcification and use of bone wax, and report early clinical outcomes.

## 2. Case presentation

### 2.1. Patient demographics and history

We present a case of a 21-year-old medically and surgically free male, who presented with chronic right anterior knee pain and discomfort, predominantly exacerbated by kneeling and jumping. He had been actively engaged in recreational basketball and was initially evaluated at the age of 15, when he was diagnosed with SLJS. Over the following 6 years, he was managed conservatively with activity modification, physiotherapy, and intermittent bracing, which led to only partial symptomatic relief.

His primary complaint remained localized discomfort over the inferior pole of the patella, particularly during activities placing mechanical stress on the extensor mechanism. There was no history of joint instability, locking, or giving way. Additionally, the patient reported the gradual development of a firm, prominent mass below the patella, which had become increasingly symptomatic over time.

Differential diagnoses considered included Osgood–Schlatter disease, patellar tendinopathy, and symptomatic bipartite patella; however, the lesion’s location, imaging characteristics, and the patient’s known diagnosis supported progression of SLJS.

### 2.2. Clinical evaluation

**Figure 1.**
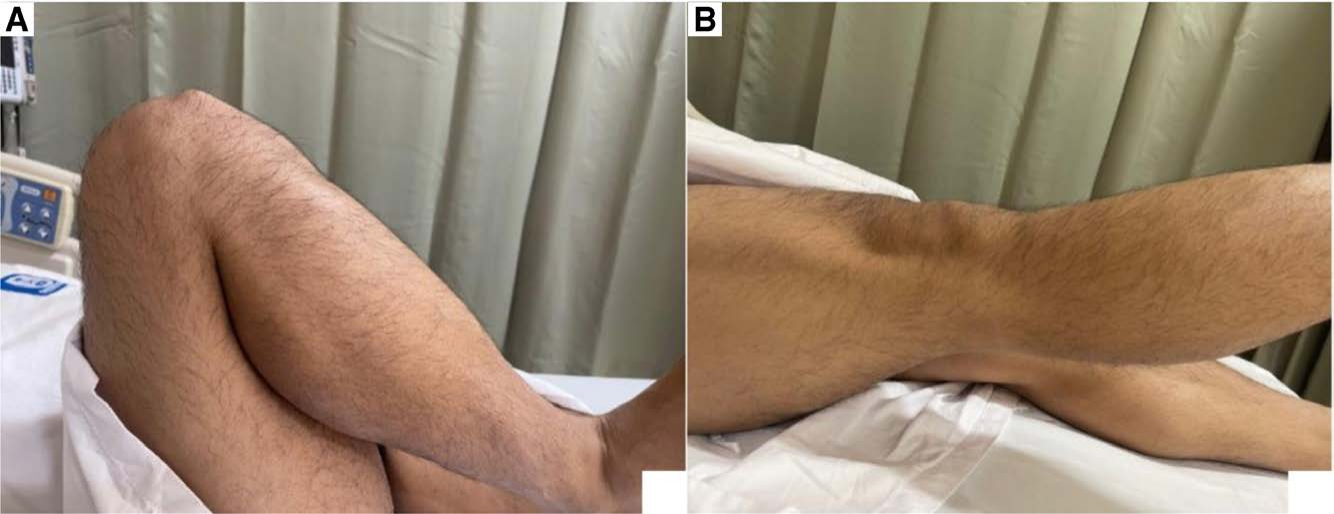
(A) Right knee in in full flexion showing inferior patella lump becoming more prominent. (B) Right knee in full extension.

On physical examination, the patient appeared fit and healthy for his age. A visible, firm lump was noted just distal to the inferior pole of the patella. The lump was minimally tender to deep palpation, mobile with patellar motion, and not associated with erythema, warmth, or effusion (Fig. 1A). There was no joint line tenderness, and the patient maintained a full, pain-free range of motion in the right knee (Fig. 1A and B). The extensor mechanism was intact, ligamentous testing was stable, and distal neuro-vascular status was preserved. There were no constitutional symptoms. The patient reported no regular medications and was only allergic to penicillin. No significant laboratory abnormalities were noted during admission.

### 2.3. Imaging findings

**Figure 2.**
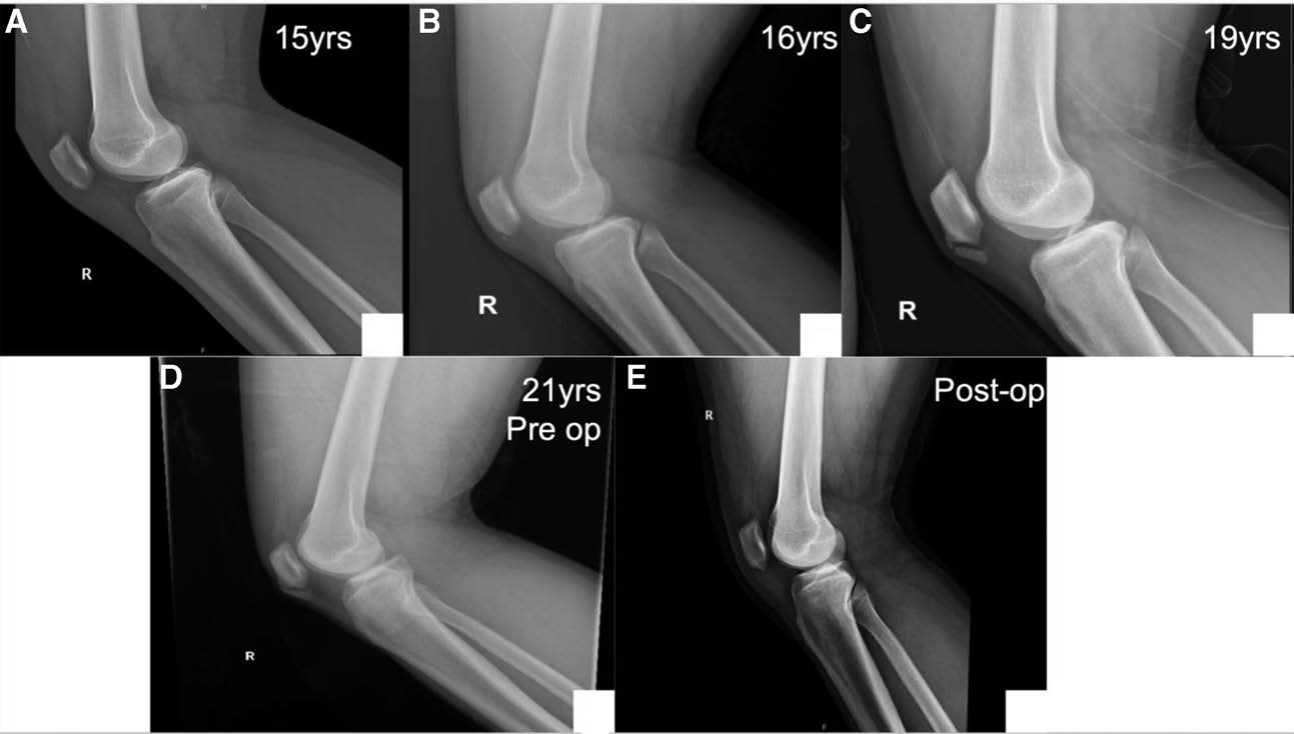
(A) Irregular calcification in the lower pole of the patella (Stage II). (B) Progressive coalescence of the small calcifications (Stage III). (C and D) Calcified ossicle, independent of the patella (Stage IV-B).

**Figure 3.**
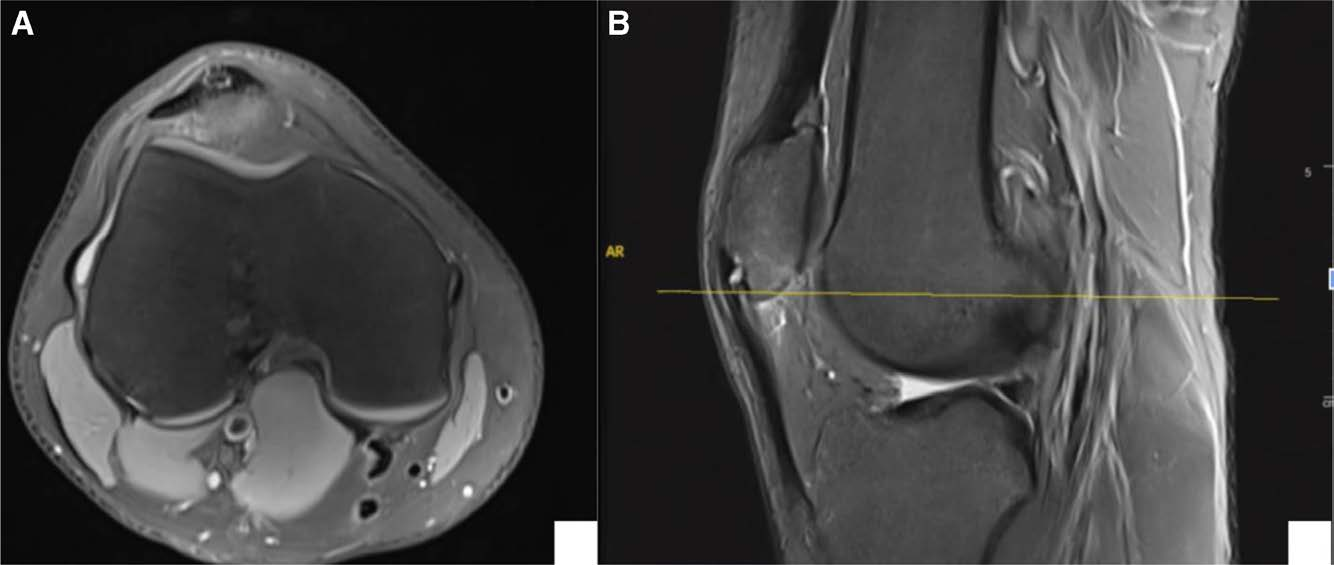
Early MRI of the knee demonstrating a Sinding-Larsen–Johansson lesion at the inferior pole of the patella. (A) Axial T2-weighted image showing focal bone marrow edema and irregularity at the patellar attachment of the patellar tendon. (B) Sagittal T2-weighted image highlighting the cortical irregularity, subchondral edema, and associated soft tissue changes consistent with osteochondrosis. MRI = magnetic resonance imaging.

**Figure 4.**
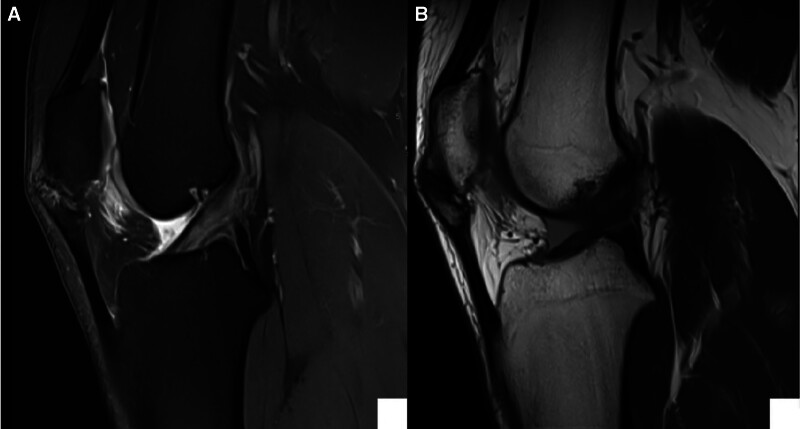
Follow-up MRI of the knee performed 3 years after the initial study, demonstrating progression of the Sinding-Larsen–Johansson lesion at the inferior pole of the patella. (A) Sagittal T2-weighted image showing persistent cortical irregularity with increased fragmentation and surrounding marrow signal changes. (B) Sagittal T1-weighted image highlighting sclerosis and chronic bony remodeling at the patellar tendon insertion site, consistent with long-standing osteochondrosis. MRI = magnetic resonance imaging.

**Figure 5.**
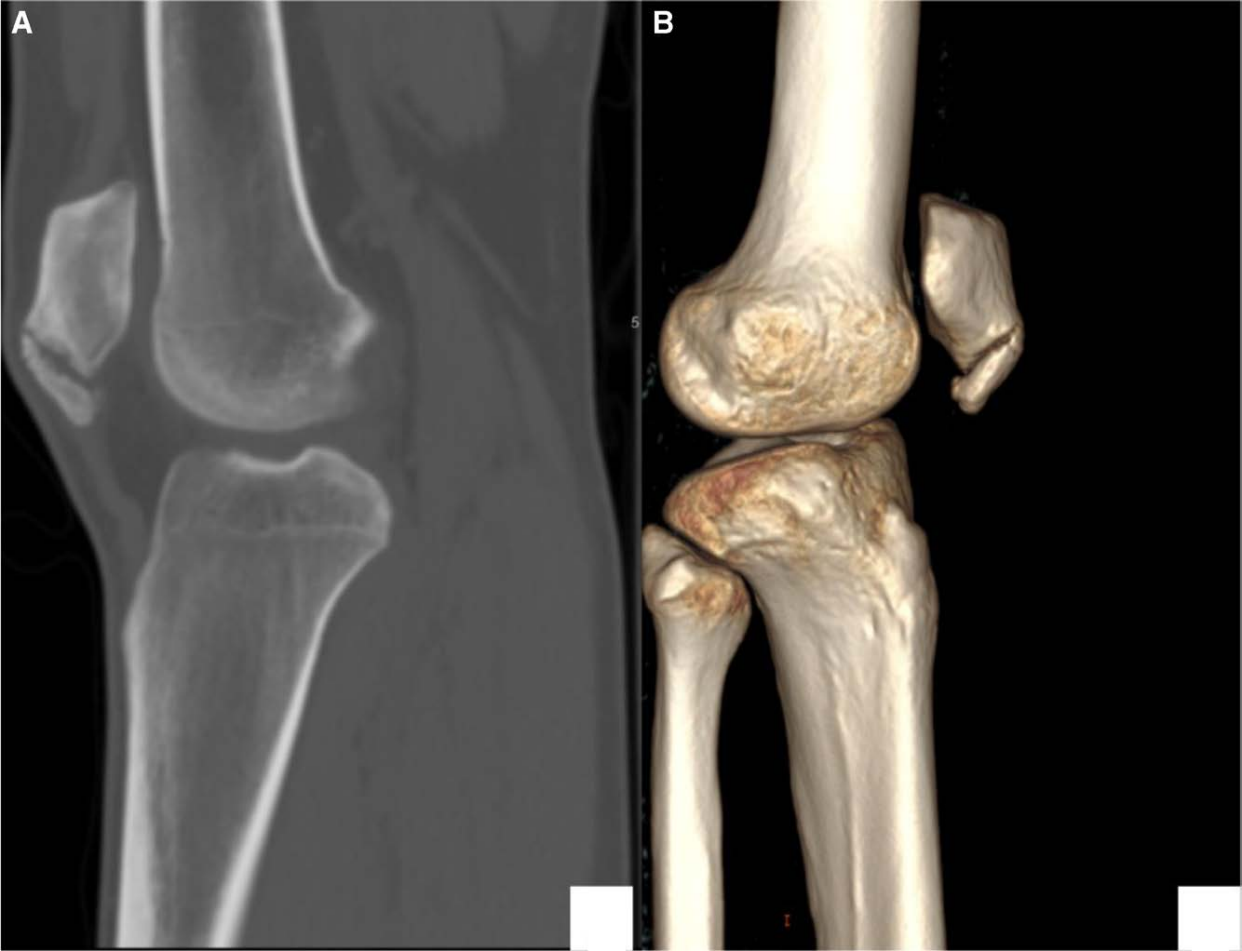
CT of the knee demonstrating a chronic Sinding-Larsen–Johansson lesion. (A) Sagittal CT image showing an ossified fragment at the inferior pole of the patella with cortical irregularity and sclerosis. (B) Three-dimensional CT reconstruction illustrating the bony fragment in relation to the patella and tibial tuberosity. CT = computed tomography.

- X-rays: since age of 15, follow-up radiographs demonstrated initially irregular calcification in the lower pole of the patella (Stage II), then coalescence of the calcific changes started to appear at the distal pole (Stage III). Finally, a distinct ossicle formation separate from the patella (Stage IV-B) (Fig. 2).
- Magnetic resonance imaging (MRI) (January 2021): revealed mild thickening and signal alterations at the patellar tendon origin, with a small ossicle and subtle bone marrow edema, suggestive of early-stage SLJS (Fig. 3).
- MRI (February 2024): showed interval progression, with increased tendon thickening, a larger ossified fragment, and more prominent infrapatellar fat pad edema (features indicative of chronic mechanical stress and impingement (Fig. 4)).
- Computed tomography scan (2024): confirmed a well-defined osteochondrosis originating from the inferior pole of the patella, with minimal separation. No evidence of acute fracture or joint involvement was observed (Fig. 5).

### 2.4. Surgical indication and procedure

Given the patient’s persistent pain, mechanical irritation, and failure of conservative therapy over several years, surgical intervention was offered, and the patient was keen to be surgically relieved from his symptomatic right knee. The goal was to excise the osteochondrosis at the inferior patellar pole and preserve the extensor mechanism’s integrity.

**Figure 6.**
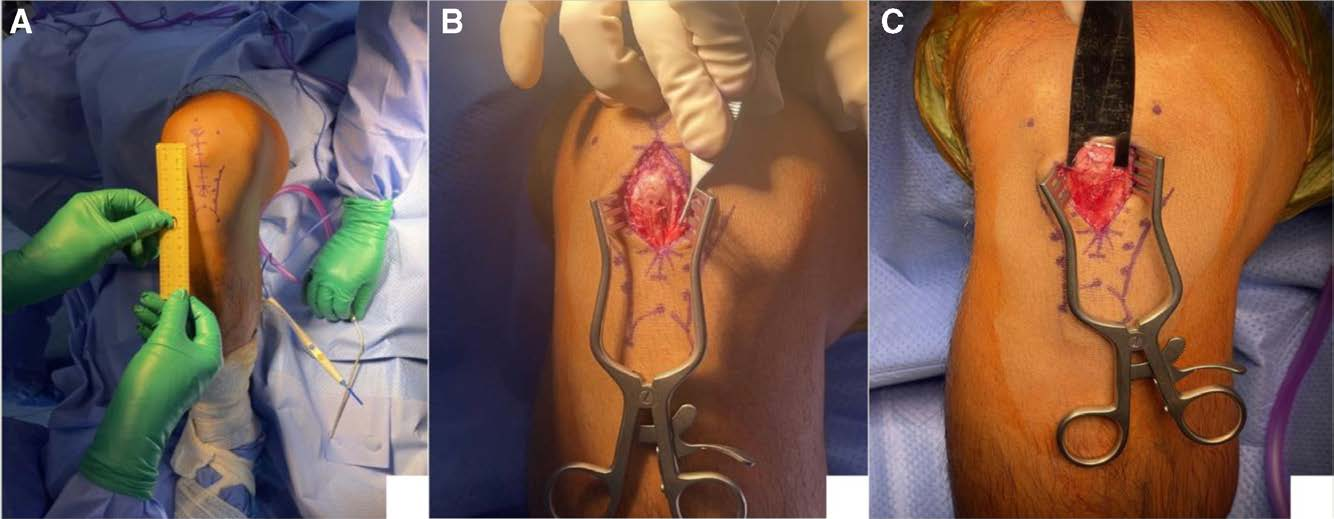
A 6 cm anterior midline incision was made over the inferior patellar prominence (A), exposure (B), and osteotomy (C).

Under general anesthesia, combined with adductor and genicular nerve blocks for analgesia, the patient was positioned supine and a non-sterile tourniquet was utilized. Standard surgical prepping and draping were performed for the right lower extremity. Landmarks, including the patella borders and tibial tuberosity, were identified. A 6 cm anterior midline incision was made over the inferior patellar prominence (Fig. 6A). Skin and subcutaneous tissue sharply incised in line with skin incision, patellar tendon paratenon identified and incised in the mid-coronal plane (Fig. 6B).

**Figure 7.**
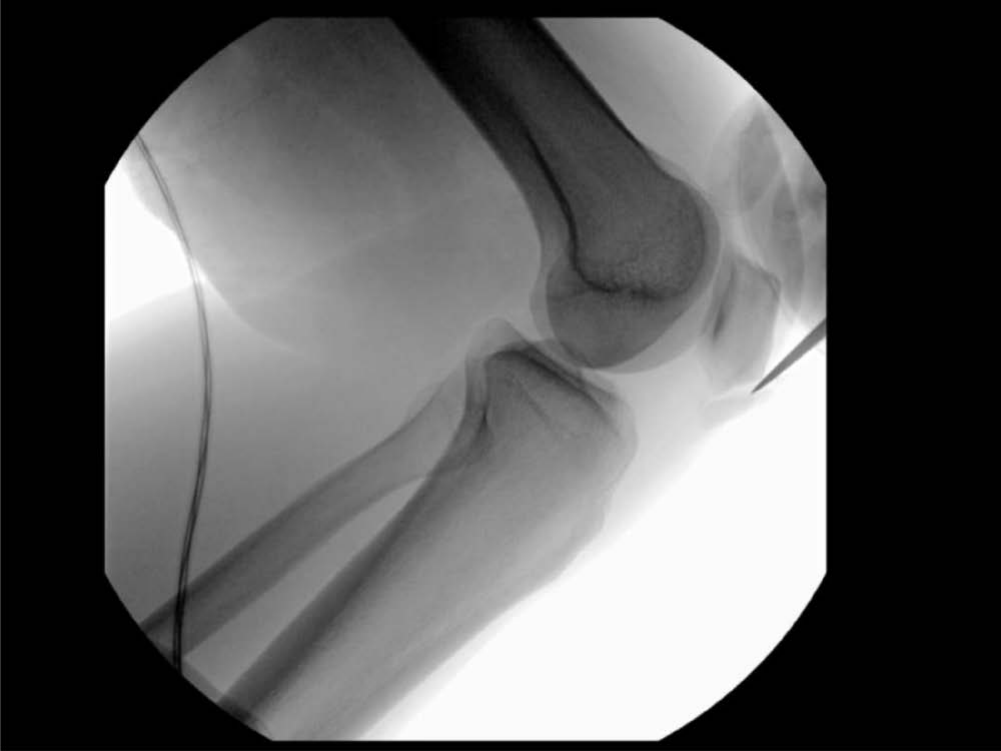
Intraoperative fluoroscopic lateral view of the knee demonstrating surgical resection of a Sinding-Larsen–Johansson lesion from the inferior pole of the patella.

Next, a large, ossified mass arising from the inferior patellar pole was identified and found to be connected by a fibro-cartilaginous synchondrosis. Under fluoroscopic guidance, the osteochondrosis was released at its superior margin using an osteotome and carefully resected. (Fig. 6C, Fig. 7).

**Figure 8.**
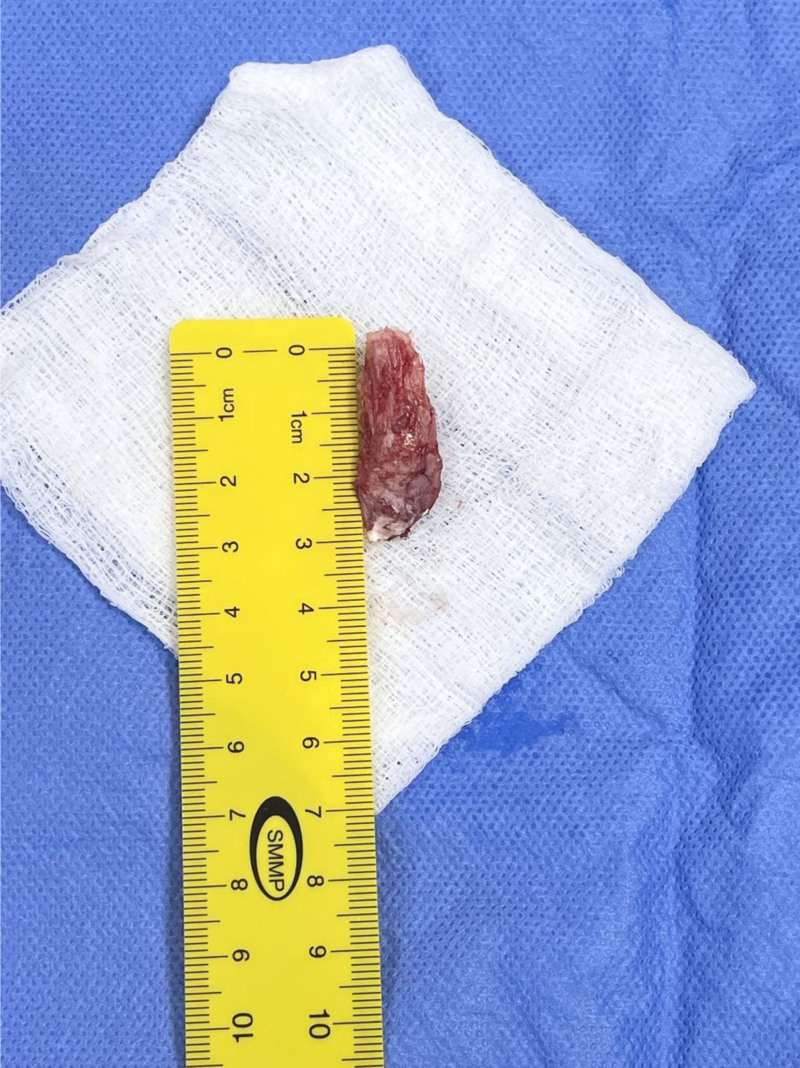
Excised Sinding-Larsen–Johansson lesion, measuring approximately 3 cm in length and 1.5 cm in width.

Minimal disruption of the patellar tendon was noted, involving <15% of its fibers. Bone wax was applied to bleeding cancellous surfaces along the remainder of the interior pole of patella, and the tendon defect was repaired side to side using 0 Vicryl sutures in a figure-of-8 configuration. Layered closure was performed, with care to repair the paratenon. The excised osteochondrosis measured around 3cm long and 1.5 cm wide (Fig. 8).

The patient was discharged on the 1st postoperative day with instructions for full weight bearing on the affected extremity as well as early passive and active knee motion limited to 90° of flexion for the 1st 2 weeks, then gradually advanced to full range of motion by 4 weeks post-operatively. At 12 weeks post-operatively, patient regained full strength and range of motion, with no mechanical symptoms or functional limitations. Patient mentioned complete resolution of pre-operative complaints of pain and discomfort. Follow-up radiographs confirmed complete removal of the ossicle with no recurrence or residual deformity. No complications were observed through out the follow-up period.

### 2.5. Postoperative care & rehabilitation

**Figure 9.**
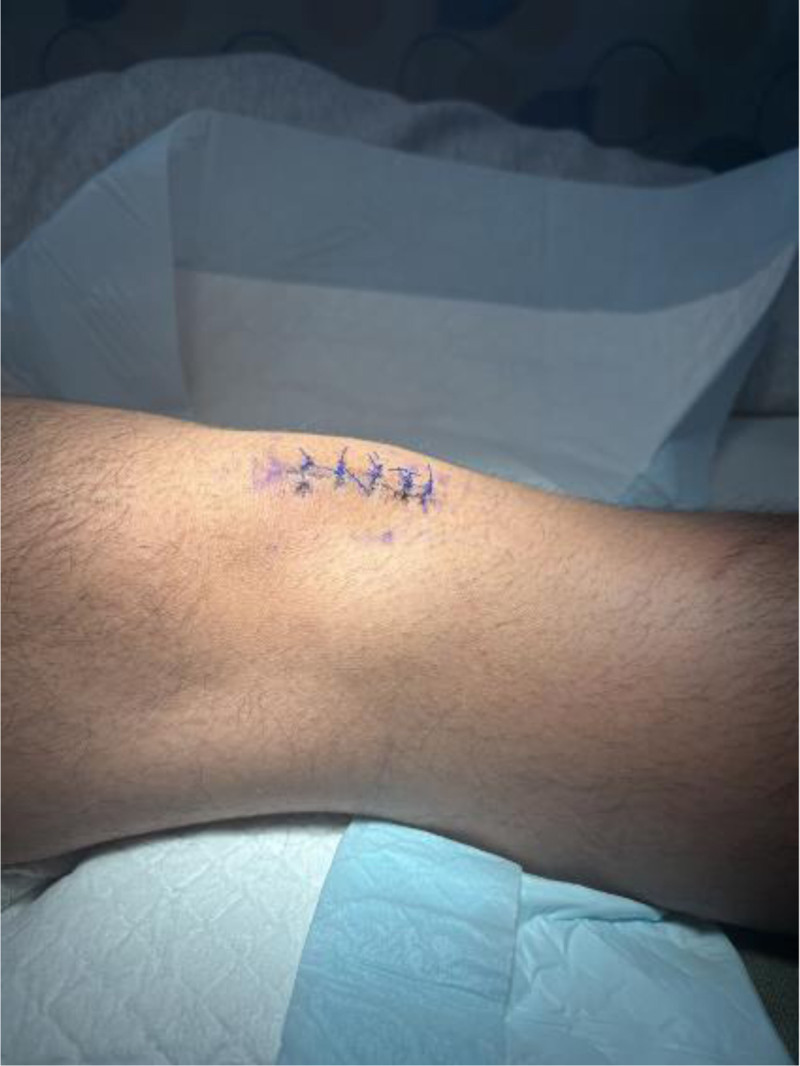
Healed surgical incision at 2 weeks post-operatively.

Two weeks post-excision of the right inferior pole of the patella inferior pole ossicle, the surgical incision had healed adequately, and sutures were removed (Fig. 9). At this stage, the patient exhibited active knee range of motion from 0° to 110°, with an intact straight leg raise against resistance. Rehabilitation focused on gradual advancement of range of motion as tolerated, with no specific restrictions. Quadriceps activation and progressive strengthening exercises were initiated, supplemented with cryotherapy as needed for comfort.

### 2.6. Follow-up and outcome

Histopathological analysis demonstrated mature bone trabeculae with adjacent fibrosis and focal vascular proliferation, consistent with a benign chronic process. At 12 weeks post-operatively, the patient had returned to normal daily activities, attained full knee range of motion, and could kneel without pain or discomfort. The clinical examination indicated stable knee function, devoid of tenderness or mechanical symptoms, and the patient expressed full satisfaction with the outcome.

## 3. Discussion

SLJS is an osteochondrosis affecting the inferior pole of the patella, primarily observed in adolescents during phases of rapid growth and heightened physical activity. This condition is classified within the spectrum of traction apophysitis resulting from recurrent tension at the attachment site of the patellar tendon in the developing skeleton. This recurrent stress results in microtrauma, inflammation, and, in certain instances, fragmentation or ossification at the distal patellar pole.^[4,6,7]^

The syndrome exhibits a pathomechanical mechanism similar to that of Osgood–Schlatter disease, characterized by noncontact traction injuries resulting from athletic activity such as jumping, sprinting, or kicking.^[8]^ In the early stages, SLJS manifests as discomfort, edema, and soreness at the inferior pole of the patella, usually absent of a singular traumatic incident. Imaging techniques, including ultrasound and MRI, may demonstrate cartilage swelling, soft tissue edema, and fragmentation of the ossification center.^[6,7]^

Medlar and Lyne proposed a 4-stage classification system for SLJS based on radiographic findings.^[4]^

- **Stage I:** normal-appearing patella despite symptoms.
- **Stage II:** irregular calcifications at the distal pole of the patella.
- **Stage III:** coalescence of the calcific changes into the distal pole.
- **Stage IV-A:** reattachment and healing of the ossification with the patella.
- **Stage IV-B:** formation of a separate ossicle, distinct from the patella, often symptomatic and visible on imaging.

These stages illustrate the transition from acute inflammation to the risk of persistent mechanical impingement if healing fails to transpire. In most instances, SLJS is self-limiting and resolves with conservative management (rest, activity moderation, physical therapy). Nevertheless, ongoing ossicle development accompanied by mechanical complaints may require surgical intervention.^[9]^

SLJS is typically a self-limiting osteochondrosis that predominantly impacts active adolescents and exhibits favorable outcomes with nonoperative interventions. The majority of cases are treated conservatively by a mix of activity reduction, physiotherapy, short-term immobilization, and analgesics. Immobilization in a cylinder cast or brace for several weeks helps mitigate symptoms during acute periods, especially when discomfort or extension lag is evident.^[8]^ Alassaf et al and Iwamoto et al highlighted that most patients recuperate within 4 to 14 weeks and resume normal activities without lingering effects.^[8]^ Sonographic and MRI data can aid in distinguishing SLJS from more severe diseases, such as sleeve fractures, hence informing noninvasive therapy options.^[7]^

Surgical option for SLJS is infrequently warranted and is reserved for chronic, refractory cases with enduring symptoms unresponsive to extended conservative treatment. In a documented adult example, arthroscopic excision of a problematic ossicle resulted in total symptom relief and reinstatement in professional sports, underscoring that surgical interventions may be contemplated in extraordinary circumstances.^[8]^ Furthermore, a case presented by Schmidt-Hebbel et al described the progression from conservatively managed SLJS to a patellar sleeve avulsion (PSA) fracture, necessitating surgical intervention with transosseous high-strength tape fixation. This uncommon consequence illustrates that, in certain instances, chronic SLJS may lead to structural failure, requiring surgical intervention.^[9]^

Kajetanek et al documented a case involving a 29-year-old professional handball player suffering from recurrent anterior knee pain attributed to SLJS, which was refractory to conservative treatment. The arthroscopic removal of the ossicle led to complete symptom relief and a return to sports within weeks.^[10]^ Nelissen et al reported on a 12-year-old soccer player with Stage IV-B SLJS, who improved following modifications to sports loading; however, they indicated that surgical intervention might have been necessary if symptoms had continued.^[11]^

The most significant consequence noted is the advancement to PSA fracture, as emphasized by Schmidt-Hebbel et al. A chronic SLJS instance progressed to a PSA, necessitating surgical fixation with transosseous sutures and high-strength tape, indicating that prolonged apophyseal stress may lead to structural failure.^[9]^

The authors elected an open approach for this patient based on the relatively large ossicle size, its fibro-cartilaginous attachment to the inferior pole, and proximity to patellar tendon fibers, where direct visualization was preferred to ensure complete excision with minimal tendon disruption. In addition, surgeon preference and the relative ease and reliability of the open technique in this anatomical setting contributed to the decision.

Bone wax is extensively utilized as a mechanical hemostatic agent in orthopedic and spinal surgeries to manage hemorrhage from cancellous bone surfaces. In SLJS surgery, especially during ossicle excision or distal pole patellar debridement, bone wax can ensure good hemostasis and preserve a clear surgical field.^[12]^

In our case, bone wax was applied selectively to the exposed cancellous surface of the inferior patellar pole following ossicle excision to achieve effective local hemostasis.^[13]^ The inferior pole contains highly vascular cancellous bone, and bleeding in this region may obscure the operative field and contribute to postoperative hematoma formation or soft-tissue irritation along the patellar tendon. Using bone wax allowed for a clear surgical field and minimized the risk of postoperative reactive inflammation due to hematoma.^[12]^ Although bone wax can theoretically delay osseous remodeling, this was not a clinical concern in this setting, as no bony healing or union was intended at the excision site.^[12]^ Its application therefore aligned with the surgical objective of controlled resection with minimal tendon disruption and smooth postoperative recovery.

This case demonstrates that SLJS, while classically self-limiting in adolescents, can persist into early adulthood and result in symptomatic ossicle formation that fails conservative therapy. It highlights the need for clinicians to recognize the small subset of patients in whom chronic pain, functional limitation, or mechanical irritation necessitates surgical intervention.

A limitation of this report is the absence of validated patient-reported outcome measures pre- and post-operatively, which would have provided more objective assessment of clinical improvement. Instead, follow-up was based on clinical examination and patient-reported symptoms, which demonstrated full, pain-free knee range of motion, return to activity, and high patient satisfaction.

## 4. Conclusion

This case illustrates that SLJS, although typically a self-limiting adolescent condition, can persist into adulthood and present with symptomatic ossified fragments unresponsive to conservative measures. In such rare chronic cases, surgical excision provides an effective solution, offering pain relief, restoration of full knee function, and return to normal activity with minimal risk of recurrence. Careful patient selection, preservation of the extensor mechanism, and structured rehabilitation are essential to achieving excellent outcomes and highlight the importance of considering surgery when nonoperative management fails. Future reports would benefit from incorporating validated outcome measures to further strengthen the evidence base.

## Author contributions

**Conceptualization:** Hamzah A. Alhamzah, Demah M. Benfaris, Zyad A. Aldosari, Abdulnasser A. Alwabel, Mohannad W. Awwad.

**Data curation:** Demah M. Benfaris, Zyad A. Aldosari, Abdulnasser A. Alwabel, Mohannad W. Awwad.

**Investigation:** Hamzah A. Alhamzah, Demah M. Benfaris, Zyad A. Aldosari, Abdulnasser A. Alwabel, Mohannad W. Awwad.

**Methodology:** Hamzah A. Alhamzah, Demah M. Benfaris, Zyad A. Aldosari, Abdulnasser A. Alwabel, Mohannad W. Awwad.

**Project administration:** Hamzah A. Alhamzah, Demah M. Benfaris, Zyad A. Aldosari, Abdulnasser A. Alwabel, Mohannad W. Awwad.

**Resources:** Hamzah A. Alhamzah, Demah M. Benfaris, Zyad A. Aldosari, Abdulnasser A. Alwabel, Mohannad W. Awwad.

**Software:** Hamzah A. Alhamzah, Demah M. Benfaris, Zyad A. Aldosari, Abdulnasser A. Alwabel, Mohannad W. Awwad.

**Supervision:** Hamzah A Alhamzah, Demah M. Benfaris, Zyad A. Aldosari, Abdulnasser A. Alwabel.

**Validation:** Hamzah A. Alhamzah, Demah M. Benfaris, Zyad A. Aldosari, Abdulnasser A. Alwabel, Mohannad W. Awwad.

**Visualization:** Hamzah A. Alhamzah, Demah M. Benfaris, Zyad A. Aldosari, Abdulnasser A. Alwabel, Mohannad W. Awwad.

**Writing – original draft:** Hamzah A. Alhamzah, Demah M. Benfaris, Zyad A. Aldosari, Abdulnasser A. Alwabel, Mohannad W. Awwad.

**Writing – review & editing:** Hamzah A. Alhamzah, Demah M. Benfaris, Zyad A. Aldosari, Abdulnasser A. Alwabel, Mohannad W. Awwad.

## References

[1] Kuehnast M Mahomed N Mistry B. Sinding-Larsen–Johansson syndrome. South African J Child Health. 2012;6:90.

[2] Sinding-Lorsen CM. A hitherto unknown affection of the patella in children. Acta Radiol. 2016;57:e42–6. 26966263 10.1177/0284185116631662

[3] Ziskin EK Carter KK Diaz-Parker S Glick BH. Anterior knee pain in young athletes: Osgood Schlatter or Sinding-Larsen–Johansson disease? JAAPA. 2023;36:1–3. 10.1097/01.JAA.0000977724.84515.ff 37751269

[4] Medlar RC Lyne ED. Sinding-Larsen–Johansson disease. Its etiology and natural history. J Bone Joint Surg Am. 1978;60:1113–6. 721864

[5] Aziz MA Radzi D Hanifah RA. Case report: a rare case of Sinding Larsen–Johansson syndrome in adult. Malaysian J Movement Health Exercise. 2020;9:17.

[6] De Flaviis L Nessi R Scaglione P Balconi G Albisetti W Derchi LE. Ultrasonic diagnosis of Osgood–Schlatter and Sinding-Larsen–Johansson diseases of the knee. Skeletal Radiol. 1989;18:193–7. 2665105 10.1007/BF00360969

[7] Peace KA Lee JC Healy J. Imaging the infrapatellar tendon in the elite athlete. Clin Radiol. 2006;61:570–8. 16784942 10.1016/j.crad.2006.02.005

[8] Alassaf N. Acute presentation of Sinding-Larsen–Johansson disease simulating patella sleeve fracture: a case report. SAGE Open Med Case Rep. 2018;6:2050313X18799242. 10.1177/2050313X18799242 PMC6131294 30210798

[9] Schmidt-Hebbel A Eggers F Schütte V Achtnich A Imhoff AB. Patellar sleeve avulsion fracture in a patient with Sinding-Larsen–Johansson syndrome: a case report. BMC Musculoskelet Disord. 2020;21:267. 32326930 10.1186/s12891-020-03297-z PMC7181494

[10] Kajetanek C Thaunat M Guimaraes T Carnesecchi O Daggett M Sonnery-Cottet B. Arthroscopic treatment of painful Sinding-Larsen–Johansson syndrome in a professional handball player. Orthop Traumatol Surg Res. 2016;102:677–80. 27450859 10.1016/j.otsr.2016.05.011

[11] Nelissen RG Rosendaal FR. A young soccer player with a painful patella. Ned Tijdschr Geneeskd. 2012;156:A3319. 22784597

[12] Inoue T Joko M Saito F. Bone wax technique for full-endoscopic lumbar laminotomy. J Spine Surg. 2023;9:98–101. 37038418 10.21037/jss-22-64 PMC10082434

[13] Amal I Soebroto H Puruhito. Comparison of bone wax and chitosan usage on post-sternotomy bone healing. Asian Cardiovasc Thorac Ann. 2020;29:203–7. 33353370 10.1177/0218492320984097

